# Mitochondriopathies as a Clue to Systemic Disorders—Analytical Tools and Mitigating Measures in Context of Predictive, Preventive, and Personalized (3P) Medicine

**DOI:** 10.3390/ijms22042007

**Published:** 2021-02-18

**Authors:** Alena Liskova, Marek Samec, Lenka Koklesova, Erik Kudela, Peter Kubatka, Olga Golubnitschaja

**Affiliations:** 1 Clinic of Obstetrics and Gynecology, Jessenius Faculty of Medicine, Comenius University in Bratislava, 036 01 Martin, Slovakia; liskova80@uniba.sk (A.L.); marek.samec@uniba.sk (M.S.); koklesova5@uniba.sk (L.K.); erik.kudela@uniba.sk (E.K.); 2 Department of Medical Biology, Jessenius Faculty of Medicine, Comenius University in Bratislava, 036 01 Martin, Slovakia; 3 European Association for Predictive, Preventive and Personalised Medicine, EPMA, 1160 Brussels, Belgium; 4 Predictive, Preventive and Personalised (3P) Medicine, Department of Radiation Oncology, University Hospital Bonn, Rheinische Friedrich-Wilhelms-Universität Bonn, 53127 Bonn, Germany

**Keywords:** mitochondrial function, dysfunction, injury, vicious circle, mitochondriopathy, energy metabolism, antioxidant mechanisms, ROS overproduction, ATP synthesis, oxidative damage, DNA repair, pathology, systemic disorders, tumorigenesis, cancer, apoptosis, neurodegeneration, diagnostic tools, liquid biopsy, biomarker panels, chronic inflammation, vasoconstriction, life-style, dietary habits, suboptimal health conditions, disease predisposition, individualised patient profile, multi-parametric analysis and machine learning, predictive, preventive, and personalized medicine (PPPM/3PM), health policy, socio-economic burden, COVID-19

## Abstract

The mitochondrial respiratory chain is the main site of reactive oxygen species (ROS) production in the cell. Although mitochondria possess a powerful antioxidant system, an excess of ROS cannot be completely neutralized and cumulative oxidative damage may lead to decreasing mitochondrial efficiency in energy production, as well as an increasing ROS excess, which is known to cause a critical imbalance in antioxidant/oxidant mechanisms and a “vicious circle” in mitochondrial injury. Due to insufficient energy production, chronic exposure to ROS overproduction consequently leads to the oxidative damage of life-important biomolecules, including nucleic acids, proteins, lipids, and amino acids, among others. Different forms of mitochondrial dysfunction (mitochondriopathies) may affect the brain, heart, peripheral nervous and endocrine systems, eyes, ears, gut, and kidney, among other organs. Consequently, mitochondriopathies have been proposed as an attractive diagnostic target to be investigated in any patient with unexplained progressive multisystem disorder. This review article highlights the pathomechanisms of mitochondriopathies, details advanced analytical tools, and suggests predictive approaches, targeted prevention and personalization of medical services as instrumental for the overall management of mitochondriopathy-related cascading pathologies.

## 1. Introduction

### 1.1. Mitochondria as the Life-Important Energy Supplier and Potential “Troublemaker”

Mitochondria are unique double-membrane organelles, capable of self-replicating their genome and representing the powerhouse of the cell. Mitochondria are essentially involved in numerous cellular processes including the generation of reactive oxygen species (ROS) as by-products of ATP synthesis [1]. To this end, the mitochondrial respiratory chain is the main site of ROS production in the cell [2]. Although mitochondria possess a powerful antioxidant system, an excess of ROS cannot be completely neutralized, and cumulative oxidative damage may lead to decreasing mitochondrial efficiency in energy production but increasing ROS excess [3], which is known to cause a critical imbalance in antioxidant/oxidant mechanisms [4] and a “vicious circle” in mitochondrial injury [5]. By insufficient energy production, chronic exposure to ROS overproduction consequently leads to the oxidative damage of life-important biomolecules, including nucleic acids, proteins, lipids, and amino acids, among others. Consequently, mitochondrial dysfunction is associated with accelerated aging, neurodegeneration, tumorigenesis, metabolic syndromes, and mood disorders, among others [1,6].

### 1.2. Liquid Biopsy Is Instrumental for the Paradigm Change from Reactive to Predictive, Preventive, and Personalized Medicine (PPPM/3PM)

Liquid biopsy is a non-invasive or minimally-invasive, cost-effective, painless, precise, and real-time analysis of specific biomarkers obtained from non-solid tissues (biofluids), such as blood, urine, tears, and/or cerebrospinal fluid [7,8,9,10]. In this review, we discuss the applicability of liquid biopsy to the overall management of mitochondriopathies and disorders known to be associated with mitochondrial dysfunction. Further, based on the accumulated knowledge on the pathomechanisms of mitochondrial injury, we exemplify health conditions that might be involved in the “vicious circle” of mitochondrial damage, which would allow the paradigm shift from reactive measures (treatment of clinically manifested pathologies) to the advanced concepts of predictive approach, targeted prevention, and the personalization of medical services [7,8,9,10,11,12,13,14,15,16,17,18].

### 1.3. Aim of Study

The aim of this review is to provide a comprehensive overview of the impact of mitochondrial dysfunctions and associated oxidative damage or energy production disorders in connection with the identification of new liquid biopsy biomarkers facilitating the management of selected diseases.

### 1.4. Source of Data

Data were recovered from the biomedical literature by the use of “mitochondria” and “ROS” and “ATP” and “marker“ or “liquid biopsy” or “mitochondrial syndromes” or other associated terms as either keywords or medical subject heading (MeSH) terms in searches of the PubMed bibliographic database. We emphasized the most recent scientific papers from the years 2015–2020.

## 2. Mitochondria as the Powerhouse of the Cell: Energy Production and ROS Formation Insights

Mitochondria are unique organelles, responsible for the production of energy in eukaryotic cells. Mitochondria harbor their own genetic material [19] that encodes 13 of 80 proteins that are important for the mitochondrial respiratory complexes [3]. The electron transport chain (ETC) is an essential process for the synthesis of energy through oxidative phosphorylation (OXPHOS) [19]. OXPHOS consist of five protein complexes (I–IV and ATP synthase) and two factors (coenzyme Q10 and cytochrome c) in the inner mitochondrial membrane. High energy phosphate production is performed through coupling electron transfer to proton translocation across the inner mitochondrial membrane, which results in an electrochemical gradient, producing a power force for the synthesis of ATP by ATP synthase [20].

**Figure 1:**
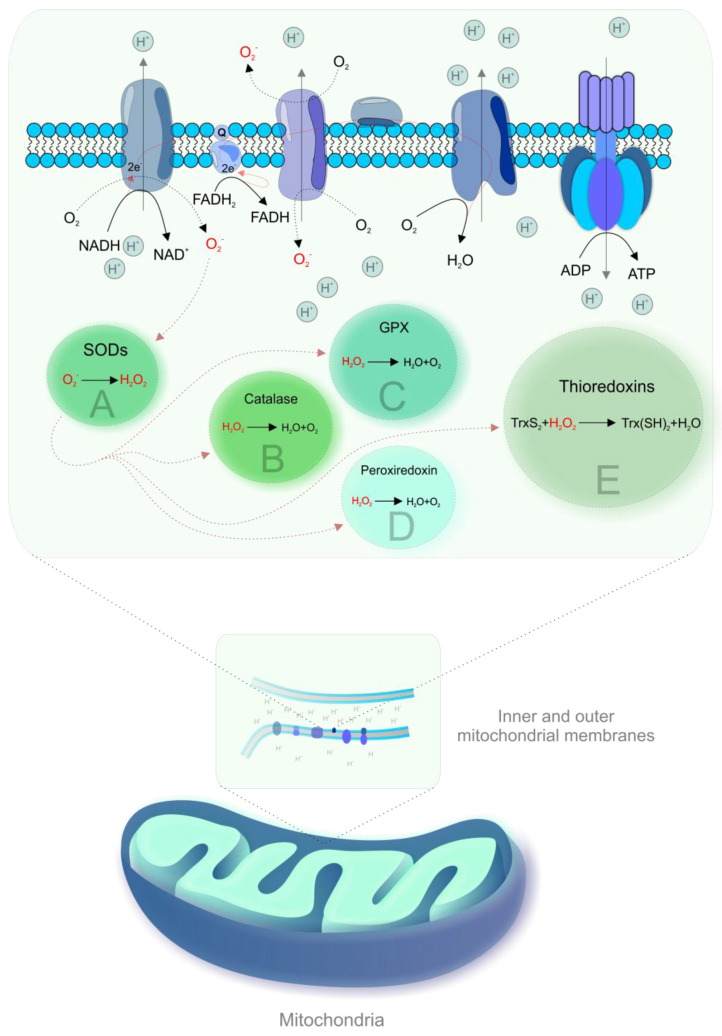
Mitochondrial reactive oxygen species (ROS) formation and antioxidant mechanisms. Complexes I and III of the electron transport chain (ETC) are the main sites of electron leakage to oxygen, yielding the superoxide anion (·O2-) [22]. The formation of ·O2- is associated with the acceptance of a unique electron by ground state oxygen and any electron transfer involving a unique electron can be susceptible to the generation of ·O2-, particularly in membranes due to the high oxygen solubility [2]. Then, ·O2- is converted to hydrogen peroxide (H_2_O_2_) by spontaneous dismutation or by an enzyme, superoxide dismutase (SOD) [23]. H_2_O_2_ is inactivated by catalase or by reaction with glutathione, catalyzed by glutathione peroxidise (GPX) [22]. Other antioxidant enzymes that contribute to ROS scavenging include peroxiredoxin and thioredoxins [24]. Highly reactive hydroxyl radical (·OH) can be produced from H_2_O_2_ in the presence of metals (iron, copper) by Haber–Weiss or Fenton reactions [22]. The generation of ROS has in an inverse association with the rate of electron transport and increases exponentially in the case of complex I or III impairment [22]. Mitochondrial dehydrogenases are also involved in ROS formation [2,3].

During OXPHOS, electrons prematurely leaking from the ETC are captured by oxygen, resulting in the generation of ROS [19,21]. Figure 1 shows mechanisms of mitochondrial ROS formation and antioxidant mechanisms.

## 3. Molecular Interplay Shifted towards Excessive ROS Formation but Diminished Energy Production—A Critical “Vicious Circle” of Mitochondrial Injury

Under physiological conditions, mitochondria serve a primary role in cellular bioenergetic activity and energy maintenance; however, their function of governing levels of mitochondria-derived ROS is no less important. The mitochondrial scavenging system, which includes SOD2, Grx2, GPx, Trx, and TrxR, is a key part of cellular redox tone. This mitochondrial antioxidant system serves to tightly control the levels of the primary ROS signaling in the mitochondrial respiration and mitochondrial dynamics [25].

However, a variety of conditions causing excessive ROS production, such as exposure to genotoxic environments, multi-factorial stress, suboptimal health conditions, and metabolic syndromes [26,27,28,29,30,31,32,33,34], may lead to unrepaired mitochondrial DNA (mtDNA) damage and concomitant functional defects in mitochondrial complexes I and III, associated with significantly increased uncontrolled formation of extremely aggressive ·O2- [3]. The arising imbalanced overproduction of ROS and consequent oxidative damage to mitochondrial structures impairs the ability of mitochondria to synthesize an appropriate amount of ATP [18] and, therefore, significantly reduces the energy resources of the cell needed to perform all the vital functions including highly energy-consuming DNA repair [35]. Molecular interplay shifted towards excessive ROS formation but diminished energy production and unrepaired DNA initiates a critical “vicious circle” in mitochondrial injury. Mitochondrial oxidative damage can also result in increased release of proteins such as cytochrome c into the cytosol by mitochondrial outer membrane permeabilization and thus activate apoptosis. Moreover, mitochondrial ROS affect the mitochondrial permeability transition pore that renders the permeability of the inner membrane to small molecules [35]. In addition, ROS induce alterations of mitochondrial Ca^2+^ homeostasis and oxidation of proteins. Peroxynitrite can inactivate key mitochondrial enzymes and trigger calcium release from mitochondria, thus affecting the energy status of the cell. An elevated level of Ca^2+^ also affects mitochondrial potential, leading to the production of ·O2- that further supports the “vicious circle” of the mitochondrial injury and development of pathologies related to mitochondriopathy [3].

In tumorigenesis, the depression of respiratory activity is an evident consequence of disruptive mtDNA mutations, further linked to enhanced generation of ROS. By acting as both mutagens and cellular mitogens, ROS contribute directly to cancer development and progression. Therefore, an impaired respiratory chain is a link between both an oxidative stress and energy failure characteristic for the mitochondrial injury on one hand, and tumorigenesis on the other hand [5]. In particular, a destabilization of complex I and secondary enhanced generation of ROS have been proposed to provoke a “vicious circle“ amplifying mitochondrial dysfunction. An excellent model to dissect the role of pathogenic, disassembling mtDNA mutations in tumor progression and their contribution to the metabolic reprogramming of cancer cells (glycolysis vs. respiration) is provided by an often underdiagnosed subset of tumors, namely, the oncocytomas, characterized by disruptive mutations of mtDNA, especially of complex I subunits. Such mutations almost completely abolish complex I activity, which slows down the Krebs cycle, favoring a high ratio of α-ketoglutarate/succinate and consequent destabilization of hypoxia inducible factor 1α (HIF1α). On the other hand, if complex I is partially defective, the levels of NAD(+) may be sufficient to implement the Krebs cycle with higher levels of intermediates that stabilize HIF1α, thus favoring tumor malignancy [5].

The “vicious circle” of mitochondrial dysfunction related to impaired mitochondrial oxidative metabolism, the uncontrolled release of excessive ROS and compromised energy production have been also exemplified by renal lipotoxicity and glomerular diseases of the kidney, which is one of the most energy-demanding organs [36].

Another prominent example of deteriorating mitochondria is cardiac ischemia-reperfusion injury (IRI). Mitochondrial injury is one of the main players in the pathology of IRI through energy stress and the overproduction of toxic ROS, leading to oxidative stress, elevated calcium, and apoptotic and necrotic cell death [37]. Therefore, various cardioprotective interventions that modulate mitochondrial stability, dynamics, and turnover, including various pharmacologic agents, specific mitochondrial antioxidants and uncouplers, and ischemic preconditioning can be considered the main strategies to protect mitochondrial and cardiovascular function and thus enhance longevity.

Finally, inflammation and mitochondrial dysfunction contribute to the pathogenesis of neurological diseases through a “vicious circle“—inflammatory mediators impair mitochondrial metabolism and defective mitochondria elicit and potentiate an inflammatory response. In detail, cytokines impede mitochondrial oxidative phosphorylation and associated ATP production and instigate mitochondrial ROS production. On the other hand, severely injured mitochondria can release their contents into the cytosol and extracellular environment and thereby amplify the inflammatory process [38]. Characteristic features of mitochondriopathies are detailed below.

## 4. Mitochondriopathies

### 4.1. Definition and Main Characteristics

Mitochondrial dysfunction (mitochondriopathy) is characterized by declined mitochondrial biogenesis, alterations in the mitochondrial membrane potential, reduced mitochondrial number, and changes in the activity of oxidative capacity due to ROS accumulation [1]. Despite the fact that an appropriate level of ROS is important for cell survival [39], there is an association between pathophysiological changes in mitochondria in aging or neurodegenerative disorders and impaired mitochondrial functions such as oxidative capacity/antioxidant imbalance, diminished OXPHOS, and reduced production of ATP [1]. Cellular dysfunction induced by a reduced ATP/ADP ratio is accompanied by increased production of ROS in mitochondria. Therefore, reduced ATP production and promoted oxidative stress triggers senescence dysfunction of highly-demanding cells such as neurons, skeletal muscle cells, and cardiac myocytes [40]. The damaging of mitochondria, the main producers of ROS, results in the accumulation of dysfunctional components caused by radicals generated by mitochondria themselves [41]. Moreover, the damaging of mtDNA after exposure to oxidative stress is worse and persist longer compared to nuclear DNA, probably due to the lack of histones and repair mechanisms in mtDNA; the occurrence of very few non-coding sequences, which increases the likelihood of the event affecting genes; and the location of mtDNA near the inner mitochondrial membrane, which is the main site of ROS generation [42]. In addition, more frequent replication of mtDNA in comparison with nuclear DNA also contributes to the higher rate of mutations. The highly polyploid state of mtDNA within each cell is associated with the occurrence of mutations that can exist in a subset of total cellular mtDNA. The replication of mtDNA molecules occurs during mitosis, but they also replicate continuously and independently of the cell cycle [43]. Interestingly, an imperfect replication process has recently been suggested to be the main source of mtDNA point mutations; DNA polymerase gamma (*POLG*) gene, which encodes DNA polymerase, which is responsible for the replication of mtDNA, could be involved in the majority of these mutagenic events [44]. Indeed, mtDNA mutator mice with exonuclease/proofreading inactive catalytic subunit of mtDNA polymerase gamma, Polg^D257A/D257A^, exerted an accumulation of mtDNA mutations in various tissues, suggesting that these may be a driving force in premature aging [45,46,47,48]. 

### 4.2. mtDNA Defects

Mitochondrial disorders are described as genetically determined syndromes associated with defective OXPHOS caused by mutations of genes encoded by mitochondrial or nuclear DNA [49]. The major cause of most mitochondrial disorders is a failure to produce an appropriate amount of ATP, which results in multisystemic disorders. Thus, extremely severe clinical manifestations are observed in highly energy-demanding tissues such as the central nervous system and skeletal and heart muscle. Nevertheless, mitochondrial dysfunction can affect any organ [50]. Mitochondrial disorders lead to major disability and also frequently result in premature death or can progress slowly in a chronic condition [49]. The clinical manifestation of mitochondrial dysfunctions suggest a specific disease phenotype and mtDNA defect [50]. Mitochondrial encephalomyopathy lactic acidosis and stroke-like episodes (MELAS) represent a clinical syndrome associated with mitochondrial point mutations (most commonly T8993G or T8993C mutations in the ATP6 gene) that is characterized by stroke-like episodes with seizures, episodes of encephalopathy, vomiting, diabetes mellitus, migraine, cardiomyopathy, cerebellar ataxia, pigmentary retinopathy, lactic acidosis, myopathy, hearing impairments, etc. [50,51,52]. A dramatic drop in mitochondrial ATP synthesis was associated with two MELAS mutations in the *tRNALeu* gene [52]. MELAS is related to deficiency in complex I/IV, resulting in ROS production. The detection of ROS in patients’ brain tissues and skin fibroblasts was performed through the detection of 8-hydroxy-2′-deoxyguanosine (8OH2′dG), decreased glutathione/oxidised glutathione (GSH/GSSH) ratio, and increased oxidative damage to lipids [53]. Other clinical syndromes associated with mtDNA mutations include neurogenic muscle weakness, ataxia and retinitis pigmentosa (NARP), myoclonic epilepsy and ragged-red fibers (MERRF), and Leber hereditary optic neuropathy (LHON) [42,50]. Although defective OXPHOS is a key characteristic of mitochondrial disorders caused by variants of mtDNA, the pathogenesis of specific variants such as MERRF extend beyond the impaired production of ATP [54]. Mitochondrial disorders related to mitochondrial mutations are usually maternally inherited or sometimes sporadic (such as single mtDNA macrodeletions) [49]. Moreover, due to the dual genetic control of nuclear and mtDNA, mutations in nuclear DNA can also contribute to mitochondrial disorders such as Leigh syndrome (LS) [50].

## 5. Liquid Biopsy Application in MELAS Management

The diagnostics of mitochondrial disorders include clinical examination and structural and functional imaging [55]. In addition, elevated lactate is usually observed in patients with MELAS and LS when compared with chronic progressive external ophthalmoplegia (CPEO) and LHON [50]. However, mitochondrial research currently offers a wide spectrum of other potential biomarkers available in cerebrospinal fluid (CSF) or other biofluids, especially blood, for the diagnosis, follow-up, and evaluation of the response to treatment of patients with mitochondrial disorders [49]. CSF represents a good source of biomarkers with diagnostic accuracy [56]; however, the disadvantage of CSF is the need for an invasive lumbar puncture that can lead to post-lumbar puncture complications [57]. Thus, the identification of usable biomarkers from more available biofluids is currently a widely discussed topic.

Fibroblast growth factor 21 (FGF21) and growth/differentiation factor 15 (GDF-15) have gained an attention as promising diagnostic biomarkers for mitochondrial disorders [58]. FGF21 is a regulator of lipid metabolism that is observed to be increased in adult mitochondrial disorders and which is also associated with the degree of mitochondrial dysfunction. GDF-15 is induced in response to defects in energy metabolism. In addition to FGF21 and GDF-15 obtained from CSF or blood [59,60], cell-free circulating mtDNA can also serve as a novel MELAS biomarker [49]. Other potential biomarkers for mitochondrial disorders include microRNA (miRNA) or small molecule reporters [55]. The utilization of small molecule reporters enables the measurement of mitochondrial function, mitochondrial-specific metabolites, and ROS generation in vivo [58]. Small molecule reporters are intravenously administered tailor-made probes that accumulate in the mitochondria of an intact organism and react with a substrate of interest. After the reaction with the substrate, probes are modified to produce an exogenous marker that can be extracted and quantified to provide inferences about the reacting substrate [55,58]. Moreover, in order to establish a marker of MELAS activity, Nukui et al. recently developed an ATP assay utilizing the luciferase luminous reaction [61]. In addition, the quantification of ROS concentration can be performed using the SNAP-tag technique based on the small molecule reporter SNAP-peroxy-green, which allows molecular imaging for H_2_O_2_ in living cells [62]. Despite so far being evaluated only preclinically, this technique can be applicable also in drug delivery and the monitoring of drug activity in mitochondria [58]. In addition, molecular genetic testing can prove mitochondrial disorders. Despite the common detection of mutations in the blood, skeletal muscle is also advisable for the detection of mitochondrial mutations [50]. Table 1 provides a detailed description of the above-discussed biomarkers that are potentially utilizable for the management of MELAS or other mitochondrial disorders.

In conclusion, the above-discussed advances in molecular medicine in the use of biomarkers for the management of mitochondrial syndromes may in the future contribute to the identification of biomarkers associated with specific pathogenic processes, including ROS or the effect on ATP, which could improve the management of patients in terms of 3P medicine.

## 6. Mitochondrial Dysfunction in Neurodegenerative Disorders

### 6.1. Oxidative Stress and Mitochondrial Dysfunction Are Central for Neurodegeneration

Oxidative stress and mitochondrial dysfunction are described as a central characteristic of brain degenerative diseases [42,64]. The brain, a structure enriched in oxidizable substrates, high oxygen demand, and relatively fewer antioxidant enzymes, contributes to the more pronounced vulnerability to oxidative stress [65]. These highly energy-demanding cells are closely associated with the mitochondria. Thus, the uptake of oxygen utilized in the mitochondrial respiratory chain allows not only the generation of energy but also ROS as by-products [66]. In addition, the brain is rich in redo-active metals including copper and iron that contribute to ROS generation. Brain cell membranes are more prone to lipid peroxidation due to their content of polyunsaturated fatty acids (PUFAs) [65]. Neurodegeneration is associated with the disruption of mitochondrial processes, including the production of ATP, membrane potential, calcium uptake, and permeability transition pore activation, which results in cell death [42,67]. Alzheimer’s disease (AD), defined as a progressive loss of memory, and Parkinson’s disease (PD), associated with impairments in movement, represent two main neurodegenerative diseases [64]. The involvement of mitochondria in neurodegenerative diseases was supported by the identification of complex I deficiency in the substantia nigra and platelet mitochondria of patients with PA and complex IV deficiency in AD. However, respiratory deficiencies could be secondary to pathogenic initiating factors, such as mutations in nuclear genes encoding non-respiratory proteins [42].

#### 6.1.1. Alzheimer’s Disease (AD)

Despite its multifactorial etiology, mitochondrial dysfunction and enhanced apoptosis are emerging hallmarks of AD [64]. The mitochondrial functions negatively affected in AD include OXPHOS, responsible for ATP production and ROS generation [22]. Oxidative damage related to abnormal marked accumulation of amyloid beta (Aβ) plaques and the deposition of neurofibrillary tangles is observed in the brains of AD patients. Biometals are suggested to play an important role in neurodegeneration due to high-affinity binding sites for copper and zinc on Aβ and its amyloid precursor protein (APP). As copper is described as a potent mediator of ·OH, it contributes to the elevated oxidative stress characteristics of the AD brain. A high concentration of copper is present in amyloid plaques [68]. Aβ plaques bind to red blood cells and impair their function through phosholipid peroxidation while these processes injure the vasculature, potentially reducing the delivery of oxygen to the brain, facilitating AD [69]. In addition, Aβ and APP levels are increased in the brains of AD patients, whereas mutations in the *APP* gene enhance Aβ production. Elevated ROS accompanied by decreased ATP was observed in SH-SY5Y cells with the mutant *APP* gene [70]. Oxidative base damage of both nuclear and mtDNA was reported in the brains of post-mortem AD patients. A mitochondrial origin of ROS in AD has been suggested, due to the high oxygen consumption of neurons and the accumulation of damaged mitochondria in AD brains [22]. Dysfunctional mitochondria produce ATP less effectively but are more effective in the production of ROS; therefore, these mitochondria are suggested to represent a main source of oxidative imbalance in AD [71]. The exacerbated ROS production, resulting in oxidative stress, increases abnormalities in neuronal cells that are often followed by apoptosis and consequent cognitive dysfunction and dementia [72].

#### 6.1.2. Parkinson’s Disease (PD)

PD is a complex, multifactorial disease affected by diverse genetic, biological, and environmental factors [73]. The progressive loss of dopaminergic neurons in the nigro-stratial system is the main feature of PD. Mitochondrial impairments and oxidative damage are described as alleged mechanisms resulting in the aggregation of α-synuclein (α-Syn) that promote dopaminergic neurotoxicity [74]. Enhanced oxidative stress in PD brains is associated with mitochondrial dysfunction, impaired iron metabolism, and elevated ROS production as a result of increased dopamine turnover [75]. PD is assumed to be triggered by mitochondrial impairment, especially through complex I dysfunction, a main entry point of the respiratory chain. Reduced activity of complex I was observed in mitochondria platelets and the substantia nigra and frontal cortex of PD patients [76]. In addition, reduced activity of complex IV was observed in post-mortem homogenates of PD patients [21]; however, a recent study revealed no significant difference in complex IV in PD [77]. Mitochondrial dysfunction involved in PD can result from impaired mitochondrial biogenesis, increased ROS generation, defects in mitophagy, dysfunction in the ETC, calcium imbalance, disrupted mitochondrial dynamics, or a combination of these [73]. The disrupted equilibrium between oxidant/antioxidant levels demonstrated in PD is suggested to be associated with the assumption that mitochondrial oxidative stress is mediated by dopamine metabolism, whereas dopamine’s ability to undergo auto-oxidation allows the production of free radicals and active quinines that interact with ROS scavengers, respiratory chain complexes, and proteins of mitophagy pathways [21]. Impaired OXPHOS leads to an increased level of ROS/RNS, which initiates a “vicious circle” that is further exacerbated by the presence of dopamine. In addition, mutations in DJ-1, a ROS scavenger, promote mitochondrial oxidative stress that acts on complex I [21]. In addition, *PINK1* deficiency leads to impairments in the respiration and inhibition of complex I and increased ROS production [67]. Moreover, the accumulation of iron in the substantia nigra of sporadic PD patients causes enhanced ROS generation and increased α-Syn aggregation. In addition, defects in other genes, such as *LRRK2* and *CHCHD2*, were associated with ROS generation in PD [73].

### 6.2. Mitochondria-Associated Liquid Biopsy Biomarker Panels in Neurodegenerative Disorders

Neurodegenerative disorders represent a serious problem for society due to the compromising of the quality of life, especially for the aging population. The traditional diagnosis of neurodegenerative diseases is not always sufficient. For example, the diagnosis of PD is feasible in case when 60%–80 % of the substantia nigra dopaminergic region is lost [75]. The diagnostics of AD are traditionally performed through the identification of specific symptoms, such as mild cognitive dementia, and labeled as probable AD; however, a definite AD diagnosis can be confirmed only after the observation of autopsy-revealed amyloid plaques or tau-based neurofibrillary tangles [78]. Nevertheless, alternatives to traditional AD diagnostics could represent new approaches such as the detection of amyloid Aβ and τ pathology through imaging of the brain [79], the development or optimization of screening tools [57], CSF assays [79], or the identification of other non-invasive biomarkers. Moreover, once diagnosed, there is a lack of non-invasive biomarkers to predict the disease progression or to achieve a prognosis [80]. Therefore, the inexistence of established biomarkers for the prediction of disease progression contributes to the challenges associated with neurodegenerative diseases. Indeed, the development of new biomarkers applicable in the management of neurodegenerative diseases is highly necessary. Although CSF is a good marker of pathological processes associated with the central nervous system, other biofluids such as blood and saliva represent a less invasive and repeatedly utilizable source of biomarkers to monitor disease progress or the responses to neurodegenerative diseases [75]. A high number of mitochondria is present in cells with a high energy demand (e.g., neurons); however, the increased risk of cardiomyopathy in AD and PD suggest systemic mitochondrial dysfunction. Systemic dysfunction can be observed in different cell types, for example in blood cells [81]. Furthermore, ROS are highly reactive, unstable, and have a short half-life, and it is thus difficult to measure them directly. Therefore, oxidized molecule products of ROS are commonly used as biomarkers, or ROS can be measured indirectly through evaluating antioxidant activity [71]. 

#### 6.2.1. Alzheimer’s Disease

An examination of bio-fluids offers a variety of biomarkers applicable in the management of AD, such as apolipoprotein E4 (ApoE4) obtained from CSF. ApoE4 is a major risk for sporadic AD and was demonstrated to impair mitochondrial function, cause oxidative stress, and damage synapses, resulting in cognitive deficits [82,83,84]. Moreover, mtDNA in CSF could serve as a useful biomarker for presymptomatic patients carrying a *PSEN1* mutation, which is associated with mitochondrial membranes and thus may affect mitochondrial functions [85,86]. *PSEN1* mutation has been recently found to be associated with increased ROS production [87]. In addition to CSF, blood cells offer several opportunities to improve AD management [79], for example by measuring oxidative stress through specific biomarkers such as specific products of lipid peroxidation (lipofuscin-like pigments) [88] or the level of H_2_O_2_, organic hydroperoxides, glutathione/glutathione disulfide ratio, glutathione transferase activity, and ATP levels [89]. Moreover, diminished cytochrome c oxidase (COX) activity observed in AD platelet mitochondria was associated with the overproduction of ROS and the underproduction of ATP [90]. Furthemore, the risk of development of late-onset AD is affected by several genetic and non-genetic factors. A familial aggregation is described in many late-onset AD cases; first degree family history of the disease, especially a parent, is the main risk factor for late-onset AD among cognitively normal individuals. Indeed, reduced COX activity in platelet mitochondria has been observed only among those cognitively normal individuals with a maternal history of late-onset AD. Therefore, this association between maternal history of late-onset AD and COX suggests a transmission through maternally inherited mtDNA [91]. In addition, increased oxidative damage has been observed in mitochondria isolated from lymphocytes of subjects with mild cognitive impairment [92]. Similarly, mitochondrial aconitase (ACO2), a Krebs cycle enzyme sensitive to oxidative damage, was found to be reduced in peripheral lymphocytes of AD and mild cognitive impairment subjects and to correlate with antioxidant protection [93]. These results highlight the potential importance of oxidative stress markers in the peripheral system to reflect brain damage and to serve as a marker for AD diagnosis, progression, or treatment [92,93]. Despite the undeniable importance of clinical testing for the identification of new potential AD biomarkers, animal studies can also provide interesting findings that can be applicable in further clinical trials. Indeed, AD-specific plaques that consist of Aβ peptides are formed from the cleavage of APP by β- and γ-secretases, whereas presenilin-1 (PS-1) is a part of the γ-secretase complex [94]. González-Domínguez et al. observed an important role of oxidative stress in AD pathogenesis, demonstrated by reduced levels of antioxidants such as uric acid, glutathione, and homocarnosine in an APP/PS1 transgenic mouse model [95]. Table 2 provides a detailed overview of the above-discussed biomarkers evaluated in AD.

Despite the above-discussed evaluation of AD biomarkers related to oxidative stress and ATP, current research on the pathogenesis of AD has produced novel insights that could be applicable for the detection of other specific biomarkers. Due to the the relevance of AD and mitochondrial dysfunction, focusing on such mechanisms could help to identify new biomarkers. Notably, blood represents a valuable source of reliable diagnostic biomarkers [96]. A decrease in endogenous basal respiration rates and in the maximal capacity of the electron transport system, and increased respiratory rates after inhibition of complex I of the electron transport system have been observed in intact platelets of AD patients. In addition, AD was associated with increased activity of complex I and decreased complex IV activity, as well as decreased plasma Q10 concentration. These results suggest the insufficiency of substrates entering OXPHOS and functional imbalance in the electron transport system, which contributes to the decreased respiration in intact platelets and mitochondrial dysfunction in the initial AD [97]. Moreover, Lunnon et al. observed altered mitochondrial genes in the blood of early AD patients [98]. Similarly, an integrated analysis of ultra-deep proteomes in the cortex, CSF, and serum revealed a mitochondrial signature in AD [99]. Furthermore, an identification of genes related to AD from blood is essential for the early diagnosis of the disease and could predict AD classification [100]. Moreover, differential expression of mRNAs related to prodrome and the progression of AD revealed that mitochondrial and ribosomal dysfunction in peripheral blood represent early signs in AD patients [101]. Finally, Perrotte et al. strengthened the potential applicability of specific oxidative stress markers as non-invasive blood-based biomarkers for AD management [102]. Conclusively, current approaches evaluating novel biomarkers for AD management could contribute to the further identification of biomarkers of specific pathological processes including mitochondrial dysfunctions and related ROS/ATP impairments, which may be used in individualized 3P medicine.

#### 6.2.2. Parkinson’s Disease

The identification and implementation of novel biomarkers is necessary for the improvement of PD management. Current research offers several biomarkers associated with mitochondrial oxidative stress or ATP that could potentially be applicable in PD, such as proteins DJ-1 and α-Syn. DJ-1 protects dopaminergic neurons against neurodegeneration in PD, as well as protecting mitochondria against oxidative stress [103] and modulating the mitochondrial response against oxidative stress. Cultured fibroblasts of a 47-year-old woman affected by a multisystem disorder characterized by progressive, early-onset parkinsonism and other impairments associated with a novel homozygous mutation in DJ-1 showed lowered ATP synthesis, high ROS levels, and reduced subunits of complex I, and abnormal markers of oxidative stress in the blood [104]. α-Syn is a component of Lewy bodies [73], the histopathological hallmark of PD described as fibrillar aggregates [105], and was identified as the first genetic familial PD gene. An increase in wild-type α-Syn and α-Syn with PD-associated mutations induced mitochondrial fragmentation and ROS production in vivo and in vitro [73]. Despite its invasive nature, CSF represents a source of DJ-1 and α-Syn as biomarkers utilizable for PD management [106,107]. However, neither DJ-1 nor α-Syn was found to be a useful plasma biomarker for PD diagnosis [106]. Products of oxidative damage of cellular components could also serve as PD biomarkers related to oxidative stress [75], such as advanced oxidized protein products [108], biopyrin measured in urine [109], the level of mitochondrial ROS in monocytes, SOD [110], glutathione peroxidase activity, oxidized glutathione, and malondialdehyde contents in blood [111]. In addition, serum uric acid has been studied in PD pathogenesis due to its role as a biomarker, as well as an antioxidant iron scavenger [112]. Current meta-analysis revealed lowered serum levels of uric acid in PD patients, whereas a further decrease was observed with disease progression [113]. Table 3 provides detailed overview of the above-discussed biomarkers potentially utilizable in the management of PD.

Due to the inexistence of accepted markers for the diagnosis and prognosis of PD, the identification of molecular signatures could improve further management of the disease and the establishment of novel markers associated with specifically monitored processes. Peripheral cells from blood share transcriptional changes that occur in the neurodegenerative brain and thus represent easily accessible tissue that could serve as a valuable source to clarify particular processes of PD [80]. Pinho et al. investigated gene expression profiling of peripheral blood and revealed that the altered expression of genes involved in various PD processes, including those associated with mitochondria, can discriminate rapid from slow PD progression [80]. The translational approach, initiated by the analysis of secretomes cultured under controlled conditions, followed by the identification of PD-related proteins with this pipeline, further translated into the evaluation of plasma samples, allows for the identification of circulating biomarkers that correlate with PD. This approach revealed a changed level of two mitochondrial-related proteins in PD patients when compared with controls [114]. In addition, primary skin fibroblasts were described as a patient-relevant model, capturing PD molecular mechanisms, and could be therefore used to identify new prognostic markers [115]. Currently, the researchers of neurodegenerative diseases are discussing the applicability of mitochondrial-derived vesicles generated by mitochondrial quality control, secreted in biofluids, in order to provide an important insight into the processes associated with PD [74,116]. The above-discussed rapid advancement in the identification of new biomarkers could potentially result in the establishment of novel markers used to evaluate the association of PD and specific processes such as mitochondrial dysfunction and the impact of ROS.

## 7. Mitochondrial Injury in Carcinogenesis: The Clue and Related Biomarker Panels

In comparison with healthy cells, cancer cells’ mitochondria can produce increased amount of ROS. Glucose deprivation in the tumor microenvironment promotes oxidative stress in tumor cells [117]. Although healthy cells primarily utilize OXPHOS to produce ATP, cancer cells preferably undergo aerobic glycolysis, even under normoxic conditions. This metabolic reprogramming of cancer cells, known as the Warburg effect, is defined by increased glucose uptake and lactate secretion, crucial for the adaptation to the hypoxic environment and hyperproliferation [118,119,120]. Cancer cells are characterized by increased production of ROS due to enhanced proliferation and glycolytic activity. Excessive ROS production in cancer cells exerts beneficial effects through promoting proliferation, aberrant metabolic activities, and angiogenesis. Tumorigensis can be promoted by oxidative stress through the accumulation of mutations [120]. Mitochondrial ROS contribute to the nuclear or mtDNA mutations and thus could promote neoplastic transformation or cancer progression [121]. In addition, elevated levels of mitochondrial ROS in cancer stem cells potentiate cancer invasiveness and metastasis through fatty acid β-oxidation, which results in the epithelial-mesenchymal transition [122]. The survival of cancer cells is highly affected by their ability to control antioxidant activity [120]. Cancer cells possess higher antioxidant capacity as a selective advantage to survive in pro-oxidant conditions, such as anti-cancer therapy [117]. Therefore, cancer cells are able to actively modify their metabolism and optimize the level of ROS and thus improve their survival [123]. Table 4 provides a brief overview of selected oxidative damage markers evaluated in lung, breast, colorectal, and prostate cancer [124,125,126,127], as these represented the most commonly diagnosed cancer types for both men and women in 2018 [128]. In conclusion, cancer offers a wide range of potential liquid biopsy markers of oxidative damage, allowing improved management of the disease.

Based on the above, although the excessive accumulation of mitochondrial ROS promotes aging, the development of neurodegenerative and metabolic disorders, and participates in mitochondrial syndromes, all of which are associated with the fatal damage of cellular structures, in cancer, due to its high adaptability and dynamism, tumor cells can use increased ROS generation to their advantage in order to survive.

## 8. Antioxidant Diet Recommendations 

Vegetable, fruit, whole grains, and medicinal plants are rich in a wide spectrum of phytochemicals. Phytochemicals can be classified into five groups including phenolics, carotenoids, alkaloids, organosulphur compounds, and nitrogen-containing compounds [129,130]. A significant benefit of phytochemicals, either isolated or in mixtures in whole plants, is associated with the wide range of biological effects in the prevention or management of various pathologies, which are based on their anti-inflammatory, antibacterial, anti-viral, anti-cancer, antioxidant, and many other health-beneficiary activities [130,131,132,133,134,135,136,137,138,139,140,141,142]. The function of phytochemicals as natural antioxidants is utilizable not only in cancer [143,144,145,146,147,148,149,150], but also in other pathologies associated with oxidative damage [151]. Despite inefficient ATP production, dysfunctional or damaged mitochondria also excessively generate ROS [97]. Mitochondrial dysfunction and associated enhanced oxidative damage represent one of the key mechanisms of aging, neurodegenerative or mitochondrial disorders [1,6,152,153]. However, the ability of phytochemicals to act as ROS scavengers or to modulate metabolic function can be utilized in the management of such diseases [154]. Flavonoids are described as important scavengers of ROS and also attenuate mitochondrial ROS formation [155]. The efficiency of numerous phytochemicals to protect cells against oxidative damage within the scope of pathologies associated with mitochondrial dysfunction has been noted, for example, in aqueous extracts of *Asparagus cochinchinensis* root [156], quercetin [157], and curcumin-loaded nanostructured lipid carrier [158] in models of AD, tyrosol [159] and *Mucuna pruriens* extract [160] in PD models, and Taurine and coenzyme Q10 in MELAS [53]. Phytochemicals also exert potent anti-cancer activity mediated through antioxidant effects [161,162].

In conclusion, a plant-based diet rich in phytochemicals offers a wide usability in the management of diseases associated with mitochondrial dysfunctions and overproduction of ROS, such as neurodegenerative or mitochondrial disorders or cancer.

## 9. Conclusions and Future Perspectives in the Context of 3P Medicine

### 9.1. Mitochondriopathies as an Attractive Diagnostic and Treatment Target

Mitochondrial injury and dysfunction (also known as mitochondriopathy) is primarily caused by inherited mutations to chromosomal and/or mtDNA and secondarily by genotoxic exogenous factors such as a toxic environment, chronic stress conditions, metabolic syndromes, etc. With an onset varying from birth to late adulthood, mitochondriopathies are represented by a spectrum of systemic diseases demonstrating a chronic progressive course with a multiorgan involvement. Although different functions including signaling, assembling, and transport can be impaired, functions of the respiratory chain are most frequently affected in mitochondriopathies, leading to imbalanced oxygen utilization, uncontrolled ROS release, and reduced energy production, acquiring the character of a “vicious circle“ (more excessive ROS, less energy production).

Different forms of mitochondriopathy may affect the brain (leucencephalopathy, calcifications, stroke-like episodes, atrophy with dementia, epilepsy, upper motor neuron signs, ataxia, extrapyramidal manifestations, fatigue) and heart, the peripheral nervous and endocrine systems, the eyes (cataract, glaucoma, pigmentary retinopathy, optic atrophy, etc.) and ears (tinnitus and deafness), gut and kidney, as well as the bone marrow [163]. Consequently, mitochondriopathies have been proposed as an attractive diagnostic target to be investigated in any patient with unexplained progressive multisystem disorder.

### 9.2. Mitochondriopathies in the Context of Prediction, Targeted Prevention, and Personalization of Medical Services

Mitochondriopathies are challenging for diagnostics and treatment, due to the broad spectrum of mitochondrial impairments. Due to an absence of causative therapies and cures for individual forms of mitochondriopathies, predictive approaches, individualized patient profiling, targeted prevention, and personalization of medical services are instrumental in the overall management of mitochondriopathies. 

Keeping in mind the above-detailed pathomechanisms, we draw attention to some specific conditions and tools to be considered at the clinic side, in order to identify predisposed individuals and to introduce targeted mitigating measures against potential mitochondrial injury and the cascaded development of related disorders.

A. Conditions
  - Multi-factorial stress conditions [26,27]
  - Genotoxic environment [28]
  - Suboptimal health conditions such as primary and secondary vasospasm leading to mitochondrial damage, due to systemic ischemic-reperfusion injury [26,31]
  - Vasoconstriction and disease predisposition to Sicca syndrome, eye disorders, “young stroke” of unclear etiology, and aggressive cancer-subtypes [15,30,32,164,165]
  - Inappropriate diet [31]
  - Metabolic syndromes such as diabetes mellitus type 2, due to multi-factorial damage to mitochondria [33] and hyperhomocysteinemia, due to chronic oxidative stress [34]
  - Chronic inflammation and predisposition to impaired healing, cancer, and neurodegeneration [166,167]
  - Autoimmune disorders such as Sjögren’s syndrome with contributing inflammatory and vascular components [168]
  - Acute infectious diseases such as COVID-19; viral infections provoke necrosis, which amplifies anti-viral immune responses, releasing damage-associated molecular patterns. Severely affected cells and tissues intrinsically secrete cell-free nucleic acids such as mtDNA. Indeed, COVID-19 patients with increased mtDNA levels are at elevated death risk and have to be intubated. Consequently, cell-free mtDNA is a potential biomarker for individualized survival status prediction in COVID-19 patients, as a model for a predictive approach under pandemic conditions [10,169,170].
B. Corresponding analytical tools
  - Application of specialized surveys [26,29,30]
  - Broad application and comprehensive analysis of liquid biopsy [7,9,10]
  - Individualized patient profiling and innovative screening programs focused on young populations [11,12,167]
  - Risk assessment, predictive, and companion diagnostics [10]
  - Multi-omics, multi-parametric analysis, and machine learning [171,172,173]
C. Mitigating measures [15,30,31,32,174]
  - Comprehensive targeted prevention
  - Life-style related expert recommendations based on individualized patient profiles
  - Dietary habits and supplements including natural scavengers and pre- and pro-biotics

Considering the enormous socio-economic burden of mitochondriopathies and associated disorders, the paradigm change from reactive medicine to PPPM strategies is strongly recommended in order to advance health policy in this area [13].

## Figures and Tables

**Table 1:** Detailed description of studies evaluating potential mitochondrial encephalomyopathy lactic acidosis and stroke-like episodes (MELAS) biomarkers.

Biomarker	Study Details	Results	Reference
FGF21 and GDF-15	Serum, CSF (case study)	Correlation between serum/CSF FGF21 and GDF-15 and MELAS vs conventional markers (lactate and pyruvate), of which the level decreased with the disease progression. FGF21 and GDF-15 were high in serum in the initial stage and greatly increased at the terminal stage of the disease	[59]
FGF21	Serum (99 adult carriers of the m.3243A. G mutation)	FGF21 in adult carriers of m.3243A >G mutation (mtDNA mutation associated with MELAS) demonstrated little value in the monitoring and prediction of of the disease course	[60]
FGF21, GDF-15, ccf-mtDNA	Blood (cohort of 123 mitochondrial patients)	Increased FGF21, GDF-15, and ccf-mtDNA in MELAS; further increased during acute events (useful biomarkers for monitoring treatment effectiveness) vs. creatine, which only differentiated severe mitochondrial patients	[49]
ROS-sensitive miRNA-9/9*	MELAS cybrids	ROS-sensitive miRNA-9/9* used to control the expression of mitochondrial tRNA-modyfing enzymes and also to be involved in molecular mechanisms of MELAS in cybrid cells. Oxidative stress-mediated induction of miRNA-9/9* post-transcriptionally negatively regulates mt-tRNA-modification enzymes. Downregulation of these enzymes by miRNA-9/9* contributes to MELAS phenotype	[63]
ATP assay (luciferase luminious reaction)		Inverse correlation between CSF ATP with disease activity	[61]
ROS quantification	Pre-clinical evaluation	SNAP-tag technique based on small molecule reporter SNAP-peroxy-green (molecular imaging for H_2_O_2_ in living cells)	[62]

Abbreviations: ATP, adenosine triphosphate; ccf-mtDNA, cell free circulating-mtDNA; CSF, cerebrospinal fluid; FGF21, fibroblast growth factor 21; GDF-15, growth/differentiation factor 15; MELAS, mitochondrial encephalomyopathy lactic acidosis, and stroke-like episodes; miRNA, microRNA; ROS, reactive oxygen species.

**Table 2:** Details of the evaluation of biomarkers potentially utilizable in Alzheimer’s disease (AD).

Biomarker (Biofluid)	Study Participants (Details)	Result	Reference
ApoE (CSF)	AD and MCI patients, and normal control from the ADNI study (*n* = 287)	Level of ApoE → discrimination between AD and normal controls	[84]
mtDNA (CSF)	Selected from a cohort of 282 subjects (AD and other cognitive disorders Unit of the Hospital Clinic of Barcelona)	Low mtDNA in presymptomatic patients with *PSEN1* mutation	[85]
Lipofuscin-like pigments (blood)	AD patients (*n* = 44) and age-matched controls (*n* = 16)	Increased lipofuscin-like pigments (productsof lipid peroxidation) in AD vs. controls	[88]
Oxidant and antioxidant metabolites (blood)	AD patients (*n* = 12), age-mached controls (*n* = 14), and young adult controls (*n* = 14)	Increased oxidative stress, hydrogen peroxide, organic hydroperoxide levels and reduced glutathione/glutathione disulfide ratio, glutathione transferase activity, and ATP in AD patients and age-matched control vs. young adult control	[89]
COX (mitochodnria isolated platelets)	AD and age-matched controls	Decrease in COX activity, diminished platelet ATP levels, and increased ROS in AD	[90]
COX	Cognitively normal (*n* = 36) individuals divided into 3 groups (parental history of late-onset AD)	Reduced COX activity in platelet mitochondria among cognitively normal individuals with maternal history of late-onset AD	[91]
Oxidative stress markers (mitochodnria isolated from lymphocytes)	Subjects with mild cognitive impairment (*n* = 12) and controls (*n* = 10)	Increased oxidative stress markers (protein carbonyls, 3-nitrotyrosine)	[92]
Mitochondrial aconitase (lymphocytes)	AD (*n* = 28), subjects with mild cognitive impairment (*n* = 22), older adults with normal cognition (*n* = 21), and younger adults with normal cognition (*n* = 19)	Mitochondrial aconitase reduced in AD and mild cognitive impairment	[93]
Antioxidants (uric acid, glutathione, homocarnosine)	In vivo model of AD (APP/PS-1 transgenic mice)	Reduced uric acid, glutathione, homocarnosine (marker of systemic oxidative stress as a hallmark of AD)	[95]

Abbreviations: AD, Alzheimer’s disease; ApoE, Apolipoprotein E4; APP, amyloid precursor protein; COX, cytochrome c oxidase; CSF, cerebrospinal fluid; MCI, mild cognitive impairment; mtDNA, mitochodnrial DNA; PS-1, presenilin-1.

**Table 3:** Details of the evaluation of biomarkers potentially utilizable in Parkinson’s disease (PD).

Biomarker	Study Participants	Result	Reference
DJ-1 (CSF)	PD patients (*n* = 43) and MSA patients (*n* = 23), and non-neurological control (*n* = 30)	CSF DJ-1 levels to distinguish MSA from PD	[107]
DJ-1 and α-Syn (blood and recently determined CSF levels)	PD patients (*n* = 126) and normal controls (*n* = 122)	Despite accessibility in CSF, DJ-1 and α-Syn are not applicable as useful plasma biomarkers for PD diagnosis	[106]
Advanced oxidized protein products (CSF, serum)	PD patients (*n* = 60) and control subjects (*n* = 45)	Higher advanced oxidized protein products (which originate as a result of the activity of free radicals) in PD patients vs negative controls	[108]
Biopyrin (urine)	PD patients (*n* = 234) and controls (*n* = 65)	Increased biopyrin (oxidative product of bilirubin) in idiopathic PD patients	[109]
ROS, SOD (blood)		Increased level of mitochondrial ROS in monocytes and reduced level of antioxidant SOD in blood	[110]
Oxidative stress markers (blood)	PD patients (*n* = 45), elderly subjetcs (*n* = 34), and adult healthy subjects (*n* = 20)	Decreased glutathione peroxidase activity, increased oxidized glutathione and malondialdehyde contents	[111]
Uric acid (blood)	Early PD patients (*n* = 42)	Lower levels of serum uric acid associated with later occurrence of mild cognitive impairments	[112]

Abbreviations: CSF, cerebrospinal fluid; PD, Parkinson’s disease; MSA, multiple system atrophy patients; ROS, reactive oxygen species; SOD, superoxide dismutase; α-Syn, α-synuclein.

**Table 4:** Liquid biopsy biomarkers associated with oxidative damage in the top four cancer types (incidence).

Cancer Type	Biofluid	Result	Reference
LC	Blood samples, LC patients (*n* = 40) and healthy controls (*n* = 40)	Higher 8OHdG in LC vs healthy control	[126]
BC	Blood samples (serum), BC patients (*n* = 35) and healthy controls (*n* = 35)	Higher MDA, GSSG in BC vs control. Lower GSH, TAC, GSH/GSSG ratio in BC vs control.	[125]
CRC	Blood samples, CRC pacients recruited into a population-based study in Germany (*n* = 3361)	Higher d-ROMs and lower TTL → poorer prognosis	[124]
PC	Blood samples, high-risk individuals (*n* = 20), healthy controls (*n* = 20)	Higher 8OHdG in high-risk subjects	[127]

Abbreviations: 8OHdG, 8-hydroxy-2′–deoxyguanosine; BC, breast cancer; CRC, colorectal cancer; d-ROMs, Diacron reactive oxygen metabolites; GSH, glutathione (total, reduced); GSSG, glutathione disulfide; LC, lung cancer; MDA, malondialdehyde; TAC, total antioxidant capacity; TTL, total thiol level; PC, prostate cancer.

## Abbreviations

O2−	Superoxide anion
OH	Hydroxyl radical
8OH2′dG	8-hydroxy-2′-deoxyguanosine
AD	Alzheimer’s disease
ADP	Adenosine diphosphate
ApoE4	Apolipoprotein E4
APP	Amyloid beta precursor protein
ATP	Adenosine triphosphate
Aβ	Amyloid beta
Ca2+	Calcium ions
CPEO	Chronic progressive external ophthalmoplegia
CSF	Cerebrospinal fluid
DNA	Deoxyribonucleic acid
ETC	Electron transport chain
FGF21	Fibroblast growth factor 21
GDF-15	Growth/differentiation factor 15
GPx	Glutathione peroxidases
GPX	Glutathione peroxidise
Grx2	Glutaredoxin-2
GSH	Glutathione
GSSH	Oxidised glutathione
H2O2	Hydrogen peroxide
HIF1α	Hypoxia inducible factor 1α
IRI	Ischemia-reperfusion injury
LHON	Leber hereditary optic neuropathy
LS	Leigh syndrome
MELAS	Mitochondrial encephalomyopathy lactic acidosis and stroke-like episodes
MERRF	Myoclonic epilepsy and ragged-red fibers
miRNA	MicroRNA
mtDNA	Mitochondrial DNA
NAD(+)	Nicotinamide adenine dinucleotide
NARP	Neurogenic muscle weakness, ataxia and retinitis pigmentosa
OXPHOS	Oxidative phosphorylation
PD	Parkinson’s disease
PPPM/3PM	Predictive, preventive, and personalized medicine
PS-1	Presenilin-1
PUFA	Polyunsaturated fatty acids
RNS	Reactive nitrogen species
ROS	Reactive oxygen species
SOD	Superoxide dismutase
SOD2	Superoxide dismutase 2
Trx	Thioredoxin
TrxR	Thioredoxin reductase
α-Syn	α-synuclein

## References

[1] Bhatti J.S., Bhatti G.K., Reddy P.H. (2017). Mitochondrial dysfunction and oxidative stress in metabolic disorders—A step towards mitochondria based therapeutic strategies. Biochim. Biophys. Acta (BBA)-Mol. Basis Dis..

[2] Bouchez C., Devin A. (2019). Mitochondrial Biogenesis and Mitochondrial Reactive Oxygen Species (ROS): A Complex Relationship Regulated by the cAMP/PKA Signaling Pathway. Cells.

[3] Guo C., Sun L., Chen X., Zhang D. (2013). Oxidative stress, mitochondrial damage and neurodegenerative diseases. Neural Regen. Res..

[4] Lee S., Tak E., Lee J., Rashid M.A., Murphy M.P., Ha J., Kim S.S. (2011). Mitochondrial H2O2 generated from electron transport chain complex I stimulates muscle differentiation. Cell Res..

[5] Gasparre G., Porcelli A.M., Lenaz G., Romeo G. (2013). Relevance of Mitochondrial Genetics and Metabolism in Cancer Development. Cold Spring Harb. Perspect. Biol..

[6] Anglin R. (2016). Mitochondrial Dysfunction in Psychiatric Illness. Can. J. Psychiatry.

[7] Liskova A., Samec M., Koklesova L., Giordano F.A., Kubatka P., Golubnitschaja O. (2020). Liquid Biopsy is Instrumental for 3PM Dimensional Solutions in Cancer Management. J. Clin. Med..

[8] Golubnitschaja O., Polivka J., Yeghiazaryan K., Berliner L. (2018). Liquid biopsy and multiparametric analysis in management of liver malignancies: New concepts of the patient stratification and prognostic approach. EPMA J..

[9] Gerner C., Costigliola V., Golubnitschaja O. (2020). Multiomic patterns in body fluids: Technological challenge with a great potential to implement the advanced paradigm of 3p medicine. Mass Spectrom. Rev..

[10] Crigna A.T., Samec M., Koklesova L., Liskova A., Giordano F.A., Kubatka P., Golubnitschaja O. (2020). Cell-free nucleic acid patterns in disease prediction and monitoring—hype or hope?. EPMA J..

[11] Golubnitschaja O., Flammer J. (2018). Individualised patient profile: Clinical utility of Flammer syndrome phenotype and general lessons for predictive, preventive and personalised medicine. EPMA J..

[12] Golubnitschaja O. (2017). Feeling cold and other underestimated symptoms in breast cancer: Anecdotes or individual profiles for advanced patient stratification?. EPMA J..

[13] Golubnitschaja O., Baban B., Boniolo G., Wang W., Bubnov R., Kapalla M., Krapfenbauer K., Mozaffari M.S., Costigliola V. (2016). Medicine in the early twenty-first century: Paradigm and anticipation—EPMA position paper 2016. EPMA J..

[14] Rocca H.-P.B.-L., Fleischhacker L., Golubnitschaja O., Heemskerk F., Helms T., Hoedemakers T., Allianses S.H., Jaarsma T., Kinkorova J., Ramaekers J. (2015). Challenges in personalised management of chronic diseases-heart failure as prominent example to advance the care process. EPMA J..

[15] Kunin A., Polivka J., Moiseeva N., Golubnitschaja O. (2018). “Dry mouth” and “Flammer” syndromes—neglected risks in adolescents and new concepts by predictive, preventive and personalised approach. EPMA J..

[16] Kudela E., Samec M., Koklesova L., Liskova A., Kubatka P., Kozubik E., Rokos T., Pribulova T., Gabonova E., Smolar M. (2020). miRNA Expression Profiles in Luminal A Breast Cancer—Implications in Biology, Prognosis, and Prediction of Response to Hormonal Treatment. Int. J. Mol. Sci..

[17] Tonhajzerova I., Olexova L.B., Jurko A., Spronck B., Jurko T., Sekaninova N., Visnovcova Z., Mestanikova A., Kudela E., Mestanik M. (2019). Novel Biomarkers of Early Atherosclerotic Changes for Personalised Prevention of Cardiovascular Disease in Cervical Cancer and Human Papillomavirus Infection. Int. J. Mol. Sci..

[18] Kudela E., Samec M., Kubatka P., Nachajova M., Laucekova Z., Liskova A., Dokus K., Biringer K., Simova D., Gabonova E. (2019). Breast Cancer in Young Women: Status Quo and Advanced Disease Management by a Predictive, Preventive, and Personalized Approach. Cancers.

[19] Poovathingal S.K., Gruber J., Halliwell B., Gunawan R. (2009). Stochastic Drift in Mitochondrial DNA Point Mutations: A Novel Perspective Ex Silico. PLoS Comput. Biol..

[20] Bergman O., Ben-Shachar D. (2016). Mitochondrial Oxidative Phosphorylation System (OXPHOS) Deficits in Schizophrenia. Can. J. Psychiatry.

[21] Grünewald A., Kumar K.R., Sue C.M. (2019). New insights into the complex role of mitochondria in Parkinson’s disease. Prog. Neurobiol..

[22] Desler C., Lillenes M.S., Tønjum T., Rasmussen L.J. (2019). The Role of Mitochondrial Dysfunction in the Progression of Alzheimer’s Disease. Curr. Med. Chem..

[23] Brookes P.S., Yoon Y., Robotham J.L., Anders M.W., Sheu S.-S. (2004). Calcium, ATP, and ROS: A mitochondrial love-hate triangle. Am. J. Physiol. Physiol..

[24] Li X., Fang P., Mai J., Choi E.T., Wang H., Yang X.-F. (2013). Targeting mitochondrial reactive oxygen species as novel therapy for inflammatory diseases and cancers. J. Hematol. Oncol..

[25] Idelchik M.D.P.S., Begley U., Begley T.J., Melendez J.A. (2017). Mitochondrial ROS control of cancer. Semin. Cancer Biol..

[26] Kunin A., Sargheini N., Birkenbihl C., Moiseeva N., Fröhlich H., Golubnitschaja O. (2020). Voice perturbations under the stress overload in young individuals: Phenotyping and suboptimal health as predictors for cascading pathologies. EPMA J..

[27] Sabel B.A., Wang J., Fähse S., Cárdenas-Morales L., Antal A. (2020). Personality and stress influence vision restoration and recovery in glaucoma and optic neuropathy following alternating current stimulation: Implications for personalized neuromodulation and rehabilitation. EPMA J..

[28] Kucera R., Pecen L., Topolcan O., Dahal A.R., Costigliola V., Giordano F.A., Golubnitschaja O. (2020). Prostate cancer management: Long-term beliefs, epidemic developments in the early twenty-first century and 3PM dimensional solutions. EPMA J..

[29] Zhu J., Ying W., Zhang L., Peng G., Chen W., Anto E.O., Wang X., Lu N., Gao S., Wu G. (2020). Psychological symptoms in Chinese nurses may be associated with predisposition to chronic disease: A cross-sectional study of suboptimal health status. EPMA J..

[30] Goncharenko V., Bubnov R., Polivka J., Zubor P., Biringer K., Bielik T., Kuhn W., Golubnitschaja O. (2019). Vaginal dryness: Individualised patient profiles, risks and mitigating measures. EPMA J..

[31] Golubnitschaja O. (2019). Flammer Syndrome: From Phenotype to Associated Pathologies, Prediction, Prevention and Personalisation.

[32] Polivka J., Pesta M., Rohan V., Celedova L., Mahajani S., Topolcan O., Golubnitschaja O. (2019). Risks associated with the stroke predisposition at young age: Facts and hypotheses in light of individualized predictive and preventive approach. EPMA J..

[33] Cebioglu M., Schild H.H., Golubnitschaja O. (2010). Cancer predisposition in diabetics: Risk factors considered for predictive diagnostics and targeted preventive measures. EPMA J..

[34] Kaplan P., Tatarkova Z., Sivonova M.K., Racay P., Lehotsky J. (2020). Homocysteine and Mitochondria in Cardiovascular and Cerebrovascular Systems. Int. J. Mol. Sci..

[35] Murphy M.P. (2008). How mitochondria produce reactive oxygen species. Biochem. J..

[36] Ge M., Fontanesi F., Merscher S., Fornoni A. (2020). The Vicious Cycle of Renal Lipotoxicity and Mitochondrial Dysfunction. Front. Physiol..

[37] Kuznetsov A.V., Javadov S., Margreiter R., Grimm M., Hagenbuchner J., Ausserlechner M.J. (2019). The Role of Mitochondria in the Mechanisms of Cardiac Ischemia-Reperfusion Injury. Antioxidants.

[38] Van Horssen J., Van Schaik P., Witte M. (2019). Inflammation and mitochondrial dysfunction: A vicious circle in neurodegenerative disorders?. Neurosci. Lett..

[39] Bae Y.S., Oh H., Rhee S.G., Yoo Y.D. (2011). Regulation of reactive oxygen species generation in cell signaling. Mol. Cells.

[40] Schutt F., Aretz S., Auffarth G.U., Kopitz J. (2012). Moderately Reduced ATP Levels Promote Oxidative Stress and Debilitate Autophagic and Phagocytic Capacities in Human RPE Cells. Investig. Ophthalmol. Vis. Sci..

[41] Brand M., Orr A., Perevoshchikova I., Quinlan C. (2013). The role of mitochondrial function and cellular bioenergetics in ageing and disease. Br. J. Dermatol..

[42] Kirkinezos I.G., Moraes C.T. (2001). Reactive oxygen species and mitochondrial diseases. Semin. Cell Dev. Biol..

[43] Lawless C., Greaves L., Reeve A.K., Turnbull D.M., Vincent A.E. (2020). The rise and rise of mitochondrial DNA mutations. Open Biol..

[44] Szczepanowska K., Trifunovic A. (2017). Origins of mtDNA mutations in ageing. Essays Biochem..

[45] Nissanka N., Bacman S.R., Plastini M.J., Moraes C.T. (2018). The mitochondrial DNA polymerase gamma degrades linear DNA fragments precluding the formation of deletions. Nat. Commun..

[46] Kujoth G.C., Hiona A., Pugh T.D., Someya S., Panzer K., Wohlgemuth S.E., Hofer T., Seo A.Y., Sullivan R., Jobling W.A. (2005). Mitochondrial DNA Mutations, Oxidative Stress, and Apoptosis in Mammalian Aging. Science.

[47] Someya S., Kujoth G.C., Kim M.-J., Hacker T.A., Vermulst M., Weindruch R., Prolla T.A. (2017). Effects of calorie restriction on the lifespan and healthspan of POLG mitochondrial mutator mice. PLoS ONE.

[48] Williams S.L., Huang J., Edwards Y.J., Ulloa R.H., Dillon L.M., Prolla T.A., Vance J.M., Moraes C.T., Züchner S. (2010). The mtDNA Mutation Spectrum of the Progeroid Polg Mutator Mouse Includes Abundant Control Region Multimers. Cell Metab..

[49] Maresca A., Del Dotto V., Romagnoli M., La Morgia C., Di Vito L., Capristo M., Valentino M.L., Carelli V., The ER-MITO Study Group (2020). Expanding and validating the biomarkers for mitochondrial diseases. J. Mol. Med..

[50] Thangaraj K., Khan N.A., Govindaraj P., Meena A.K. (2015). Mitochondrial disorders: Challenges in diagnosis & treatment. Indian J. Med Res..

[51] El-Hattab A.W., Adesina A.M., Jones J., Scaglia F. (2015). MELAS syndrome: Clinical manifestations, pathogenesis, and treatment options. Mol. Genet. Metab..

[52] Szczepanowska J., Malińska D., Wieckowski M.R., Duszyński J. (2012). Effect of mtDNA point mutations on cellular bioenergetics. Biochim. Biophys. Acta (BBA)-Gen. Subj..

[53] Hayashi G., Cortopassi G. (2015). Oxidative stress in inherited mitochondrial diseases. Free Radic. Biol. Med..

[54] Finsterer J., Zarrouk-Mahjoub S., Shoffner J.M. (2018). MERRF Classification: Implications for Diagnosis and Clinical Trials. Pediatr. Neurol..

[55] Finsterer J., Zarrouk-Mahjoub S. (2018). Biomarkers for Detecting Mitochondrial Disorders. J. Clin. Med..

[56] Hansson O., Lehmann S., Otto M., Zetterberg H., Lewczuk P. (2019). Advantages and disadvantages of the use of the CSF Amyloid β (Aβ) 42/40 ratio in the diagnosis of Alzheimer’s Disease. Alzheimer’s Res. Ther..

[57] Niemantsverdriet E., Valckx S., Bjerke M., Engelborghs S. (2017). Alzheimer’s disease CSF biomarkers: Clinical indications and rational use. Acta Neurol. Belg..

[58] Steele H.E., Horvath R., Lyon J.J., Chinnery P.F. (2017). Monitoring clinical progression with mitochondrial disease biomarkers. Brain.

[59] Matsui M., Yamadera M., Saito T., Fujimura H., Sakoda S., Koga Y. (2019). Biomarker changes associated with clinical symptoms in MELAS patient. Neurol. Clin. Neurosci..

[60] Koene S., De Laat P., Van Tienoven D.H., Vriens D., Brandt A.M., Sweep F.C.G.J., Rodenburg R.J., Donders A.R.T., Janssen M.C., Smeitink J.A.M. (2014). Serum FGF21 levels in adult m.3243A>G carriers: Clinical implications. Neurol..

[61] Nukui T., Matsui A., Niimi H., Yamamoto M., Mastuda N., Piao J.-L., Noguchi K., Kitajima I., Nakastuji Y. (2020). Cerebrospinal fluid ATP as a potential biomarker in patients with mitochondrial myopathy, encephalopathy, lactic acidosis, and stroke like episodes (MELAS). Mitochondrion.

[62] Srikun D., Albers A.E., Nam C.I., Iavarone A.T., Chang C.J. (2010). Organelle-Targetable Fluorescent Probes for Imaging Hydrogen Peroxide in Living Cells via SNAP-Tag Protein Labeling. J. Am. Chem. Soc..

[63] Meseguer S., Martínez-Zamora A., Garcia-Arumi E., Andreu A.L., Armengod M.-E. (2014). The ROS-sensitive microRNA-9/9* controls the expression of mitochondrial tRNA-modifying enzymes and is involved in the molecular mechanism of MELAS syndrome. Hum. Mol. Genet..

[64] Manoharan S., Guillemin G.J., Abiramasundari R.S., Essa M.M., Akbar M.D. (2016). The Role of Reactive Oxygen Species in the Pathogenesis of Alzheimer’s Disease, Parkinson’s Disease, and Huntington’s Disease: A Mini Review. Oxidative Med. Cell. Longev..

[65] Singh A., Kukreti R., Saso L., Kukreti S. (2019). Oxidative Stress: A Key Modulator in Neurodegenerative Diseases. Molecules.

[66] Kausar S., Wang F., Cui H. (2018). The Role of Mitochondria in Reactive Oxygen Species Generation and Its Implications for Neurodegenerative Diseases. Cells.

[67] Angelova P.R., Abramov A.Y. (2018). Role of mitochondrial ROS in the brain: From physiology to neurodegeneration. FEBS Lett..

[68] Huang W.-J., Zhang X., Chen W.-W. (2016). Role of oxidative stress in Alzheimer’s disease. Biomed. Rep..

[69] Nakagawa K., Kiko T., Miyazawa T., Sookwong P., Tsuduki T., Satoh A., Miyazawa T. (2011). Amyloid β-induced erythrocytic damage and its attenuation by carotenoids. FEBS Lett..

[70] Arrozi A.P., Shukri S.N.S., Ngah W.Z.W., Yusof Y.A.M., Damanhuri M.H.A., Jaafar F., Makpol S. (2020). Comparative Effects of Alpha- and Gamma-Tocopherol on Mitochondrial Functions in Alzheimer’s Disease In Vitro Model. Sci. Rep..

[71] Wang X., Wang W., Li L., Perry G., Lee H.-G., Zhu X. (2014). Oxidative stress and mitochondrial dysfunction in Alzheimer’s disease. Biochim. Biophys. Acta (BBA)-Mol. Basis Dis..

[72] Teixeira J.P., De Castro A.A., Soares F.V., Da Cunha E.F.F., Ramalho T.C. (2019). Future Therapeutic Perspectives into the Alzheimer’s Disease Targeting the Oxidative Stress Hypothesis. Molecules.

[73] Park J.-S., Davis R.L., Sue C.M. (2018). Mitochondrial Dysfunction in Parkinson’s Disease: New Mechanistic Insights and Therapeutic Perspectives. Curr. Neurol. Neurosci. Rep..

[74] Picca A., Guerra F., Calvani R., Bucci C., Monaco M.R.L., Bentivoglio A.R., Landi F., Bernabei R., Marzetti E. (2019). Mitochondrial-Derived Vesicles as Candidate Biomarkers in Parkinson’s Disease: Rationale, Design and Methods of the EXosomes in PArkiNson Disease (EXPAND) Study. Int. J. Mol. Sci..

[75] Bougea A. (2020). New markers in Parkinson’s disease. Adv. Clin. Chem..

[76] Marella M., Seo B.B., Yagi T., Matsuno-Yagi A. (2009). Parkinson’s disease and mitochondrial complex I: A perspective on the Ndi1 therapy. J. Bioenerg. Biomembr..

[77] Foti S.C., Hargreaves I., Carrington S., Kiely A.P., Houlden H., Holton J.L. (2019). Cerebral mitochondrial electron transport chain dysfunction in multiple system atrophy and Parkinson’s disease. Sci. Rep..

[78] Petersen R.C. (2018). How early can we diagnose Alzheimer disease (and is it sufficient)?. Neurology.

[79] Wojsiat J., Laskowska-Kaszub K., Mietelska-Porowska A., Wojda U. (2017). Search for Alzheimer’s disease biomarkers in blood cells: Hypotheses-driven approach. Biomark. Med..

[80] Pinho R., Guedes L.C., Soreq L., Lobo P.P., Mestre T., Coelho M., Rosa M.M., Gonçalves N., Wales P., Mendes T. (2016). Gene Expression Differences in Peripheral Blood of Parkinson’s Disease Patients with Distinct Progression Profiles. PLoS ONE.

[81] Gezen-Ak D., Alaylıoğlu M., Genç G., Şengül B., Keskin E., Sordu P., Güleç Z.E.K., Apaydın H., Bayram-Gürel Ç., Ulutin T. (2020). Altered Transcriptional Profile of Mitochondrial DNA-Encoded OXPHOS Subunits, Mitochondria Quality Control Genes, and Intracellular ATP Levels in Blood Samples of Patients with Parkinson’s Disease. J. Alzheimer’s Dis..

[82] Schmukler E., Solomon S., Simonovitch S., Goldshmit Y., Wolfson E., Michaelson D.M., Pinkas-Kramarski R. (2020). Altered mitochondrial dynamics and function in APOE4-expressing astrocytes. Cell Death Dis..

[83] Yin J., Nielsen M., Carcione T., Li S., Shi J. (2019). Apolipoprotein E regulates mitochondrial function through the PGC-1α-sirtuin 3 pathway. Aging.

[84] Llano D.A., Bundela S., Mudar R.A., Devanarayan V., (Adni) F.T.A.D.N.I. (2017). A multivariate predictive modeling approach reveals a novel CSF peptide signature for both Alzheimer’s Disease state classification and for predicting future disease progression. PLoS ONE.

[85] Podlesniy P., Figueiro-Silva J., Llado A., Antonell A., Sanchez-Valle R., Alcolea D., Lleo A., Molinuevo J.L., Serra N., Trullas R. (2013). Low cerebrospinal fluid concentration of mitochondrial DNA in preclinical Alzheimer disease. Ann. Neurol..

[86] Sarasija S., Norman K.R. (2018). Role of Presenilin in Mitochondrial Oxidative Stress and Neurodegeneration in Caenorhabditis elegans. Antioxidants.

[87] Raut S., Patel R., Al-Ahmad A.J. (2021). Presence of a mutation in PSEN1 or PSEN2 gene is associated with an impaired brain endothelial cell phenotype in vitro. Fluids Barriers CNS.

[88] Skoumalová A., Ivica J., Šantorová P., Topinková E., Wilhelm J. (2011). The lipid peroxidation products as possible markers of Alzheimer’s disease in blood. Exp. Gerontol..

[89] Kosenko E., Aliev G., Kaminsky Y. (2016). Relationship between chronic disturbance of 2,3-diphosphoglycerate metabolism in erythrocytes and Alzheimer disease. CNS Neurol. Disord.-Drug Targets.

[90] Cardoso S.M., Proença M., Santos S., Santana I., Oliveira C.R. (2004). Cytochrome c oxidase is decreased in Alzheimer’s disease platelets. Neurobiol. Aging.

[91] Mosconi L., de Leon M., Murray J., Lu J., Javier E., McHugh P., Swerdlow R.H. (2011). Reduced Mitochondria Cytochrome Oxidase Activity in Adult Children of Mothers with Alzheimer’s Disease. J. Alzheimer’s Dis..

[92] Sultana R., Baglioni M., Cecchetti R., Cai J., Klein J.B., Bastiani P., Ruggiero C., Mecocci P., Butterfield D.A. (2013). Lymphocyte mitochondria: Toward identification of peripheral biomarkers in the progression of Alzheimer disease. Free Radic. Biol. Med..

[93] Mangialasche F., Baglioni M., Cecchetti R., Kivipelto M., Ruggiero C., Piobbico D., Kussmaul L., Monastero R., Brancorsini S., Mecocci P. (2015). Lymphocytic Mitochondrial Aconitase Activity is Reduced in Alzheimer’s Disease and Mild Cognitive Impairment. J. Alzheimer’s Dis..

[94] Evans A.R., Gu L., Guerrero R., Robinson R.A.S. (2015). Global cPILOT analysis of the APP/PS-1 mouse liver proteome. Proteom.-Clin. Appl..

[95] González-Domínguez R., García-Barrera T., Vitorica J., Gómez-Ariza J.L. (2015). Metabolomic investigation of systemic manifestations associated with Alzheimer’s disease in the APP/PS1 transgenic mouse model. Mol. BioSyst..

[96] Mitelpunkt A., Galili T., Kozlovski T., Bregman N., Shachar N., Markus-Kalish M., Benjamini Y. (2020). Novel Alzheimer’s disease subtypes identified using a data and knowledge driven strategy. Sci. Rep..

[97] Fišar Z., Hroudová J., Hansíková H., Spáčilová J., Lelková P., Wenchich L., Jirák R., Zvěřová M. (2016). Mitochondrial Respiration in the Platelets of Patients with Alzheimer’s Disease. Curr. Alzheimer Res..

[98] Lunnon K., Keohane A., Pidsley R., Newhouse S., Riddoch-Contreras J., Thubron E.B., Devall M., Soininen H., Kłoszewska I., Mecocci P. (2017). Mitochondrial genes are altered in blood early in Alzheimer’s disease. Neurobiol. Aging.

[99] Wang H., Dey K.K., Chen P.-C., Li Y., Niu M., Cho J.-H., Wang X., Bai B., Jiao Y., Chepyala S.R. (2020). Integrated analysis of ultra-deep proteomes in cortex, cerebrospinal fluid and serum reveals a mitochondrial signature in Alzheimer’s disease. Mol. Neurodegener..

[100] Lee T., Lee H. (2020). Prediction of Alzheimer’s disease using blood gene expression data. Sci. Rep..

[101] Xue W., Li J., Fu K., Teng W. (2020). Differential Expression of mRNAs in Peripheral Blood Related to Prodrome and Progression of Alzheimer’s Disease. BioMed Res. Int..

[102] Perrotte M., Le Page A., Fournet M., Le Sayec M., Rassart É., Fulop T., Ramassamy C. (2019). Blood-based redox-signature and their association to the cognitive scores in MCI and Alzheimer’s disease patients. Free Radic. Biol. Med..

[103] Dolgacheva L.P., Berezhnov A.V., Fedotova E.I., Zinchenko V.P., Abramov A.Y. (2019). Role of DJ-1 in the mechanism of pathogenesis of Parkinson’s disease. J. Bioenerg. Biomembr..

[104] Di Nottia M., Masciullo M., Verrigni D., Petrillo S., Modoni A., Rizzo V., Di Giuda D., Rizza T., Niceta M., Torraco A. (2016). DJ-1 modulates mitochondrial response to oxidative stress: Clues from a novel diagnosis of PARK7. Clin. Genet..

[105] Wakabayashi K., Tanji K., Odagiri S., Miki Y., Mori F., Takahashi H. (2013). The Lewy Body in Parkinson’s Disease and Related Neurodegenerative Disorders. Mol. Neurobiol..

[106] Shi M., Zabetian C.P., Hancock A.M., Ginghina C., Hong Z., Yearout D., Chung K.A., Quinn J.F., Peskind E.R., Galasko D. (2010). Significance and confounders of peripheral DJ-1 and alpha-synuclein in Parkinson’s disease. Neurosci. Lett..

[107] Herbert M.K., Eeftens J.M., Aerts M.B., Esselink R.A., Bloem B.R., Kuiperij H.B., Verbeek M.M. (2014). CSF levels of DJ-1 and tau distinguish MSA patients from PD patients and controls. Park. Relat. Disord..

[108] Garcia-Moreno J.-M., De Pablos A.M., García-Sánchez M.-I., Méndez-Lucena C., Damas-Hermoso F., Rus M., Chacón J., Fernández E., Fernández-Espejo E., Rus-Hidalgo M. (2013). May Serum Levels of Advanced Oxidized Protein Products Serve as a Prognostic Marker of Disease Duration in Patients with Idiopathic Parkinson’s Disease?. Antioxid. Redox Signal..

[109] Luan H., Liu L.-F., Tang Z., Mok V.C., Li M., Cai Z. (2015). Elevated excretion of biopyrrin as a new marker for idiopathic Parkinson’s disease. Park. Relat. Disord..

[110] Smith A.M., Depp C., Ryan B.J., Johnston G.I., Alegre-Abarrategui J., Evetts S., Rolinski M., Baig F., Ruffmann C., Simon A.K. (2018). Mitochondrial dysfunction and increased glycolysis in prodromal and early Parkinson’s blood cells. Mov. Disord..

[111] Vida C., Kobayashi H., Garrido A., De Toda I.M., Carro E., Molina J.A., De La Fuente M. (2019). Lymphoproliferation Impairment and Oxidative Stress in Blood Cells from Early Parkinson’s Disease Patients. Int. J. Mol. Sci..

[112] Pellecchia M.T., Savastano R., Moccia M., Picillo M., Siano P., Erro R., Vallelunga A., Amboni M., Vitale C., Santangelo G. (2016). Lower serum uric acid is associated with mild cognitive impairment in early Parkinson’s disease: A 4-year follow-up study. J. Neural Transm..

[113] Wen M., Zhou B., Chen Y.-H., Ma Z.-L., Gou Y., Zhang C.-L., Yu W.-F., Jiao L. (2017). Serum uric acid levels in patients with Parkinson’s disease: A meta-analysis. PLoS ONE.

[114] Anjo S.I., dos Santos P.V., Rosado L., Baltazar G., Baldeiras I., Pires D., Gomes A., Januário C., Castelo-Branco M., Grãos M. (2020). A different vision of translational research in biomarker discovery: A pilot study on circulatory mitochondrial proteins as Parkinson’s disease potential biomarkers. Transl. Neurodegener..

[115] Teves J.M.Y., Bhargava V., Kirwan K.R., Corenblum M.J., Justiniano R., Wondrak G.T., Anandhan A., Flores A.J., Schipper D.A., Khalpey Z. (2018). Parkinson’s Disease Skin Fibroblasts Display Signature Alterations in Growth, Redox Homeostasis, Mitochondrial Function, and Autophagy. Front. Neurosci..

[116] Picca A., Guerra F., Calvani R., Marini F., Biancolillo A., Landi G., Beli R., Landi F., Bernabei R., Bentivoglio A.R. (2020). Mitochondrial Signatures in Circulating Extracellular Vesicles of Older Adults with Parkinson’s Disease: Results from the EXosomes in PArkiNson’s Disease (EXPAND) Study. J. Clin. Med..

[117] Movahed Z.G., Rastegari-Pouyani M., Mohammadi M.H., Mansouri K. (2019). Cancer cells change their glucose metabolism to overcome increased ROS: One step from cancer cell to cancer stem cell?. Biomed. Pharmacother..

[118] Samec M., Liskova A., Koklesova L., Samuel S.M., Zhai K., Buhrmann C., Varghese E., Abotaleb M., Qaradakhi T., Zulli A. (2020). Flavonoids against the Warburg phenotype—concepts of predictive, preventive and personalised medicine to cut the Gordian knot of cancer cell metabolism. EPMA J..

[119] Samec M., Liskova A., Koklesova L., Mersakova S., Strnadel J., Kajo K., Pec M., Zhai K., Smejkal K., Mirzaei S. (2021). Flavonoids Targeting HIF-1: Implications on Cancer Metabolism. Cancers.

[120] Gwangwa M.V., Joubert A.M., Visagie M.H. (2018). Crosstalk between the Warburg effect, redox regulation and autophagy induction in tumourigenesis. Cell. Mol. Biol. Lett..

[121] Sabharwal S.S., Schumacker P.T. (2014). Mitochondrial ROS in cancer: Initiators, amplifiers or an Achilles’ heel?. Nat. Rev. Cancer.

[122] Wang C., Shao L., Pan C., Ye J., Ding Z., Wu J., Du Q., Ren Y., Zhu C. (2019). Elevated level of mitochondrial reactive oxygen species via fatty acid β-oxidation in cancer stem cells promotes cancer metastasis by inducing epithelial–mesenchymal transition. Stem Cell Res. Ther..

[123] Rodic S., Vincent M.D. (2018). Reactive oxygen species (ROS) are a key determinant of cancer’s metabolic phenotype. Int. J. Cancer.

[124] Boakye D., Jansen L., Schöttker B., Jansen E.H.J.M., Schneider M., Halama N., Gào X., Chang-Claude J., Hoffmeister M., Brenner H. (2020). Blood markers of oxidative stress are strongly associated with poorer prognosis in colorectal cancer patients. Int. J. Cancer.

[125] El-Soud M.R.A., Hewala T.I.M. (2019). The clinical significance of serum oxidative stress biomarkers in breast cancer females. Med Res. J..

[126] Antošová M., Bencova A., Mikolka P., Košútová P., Mokra D., Rozborilová E. (2015). The markers of oxidative stress in patient with lung cancer. Eur. Respir. J..

[127] Shukla S., Srivastava J.K., Shankar E., Kanwal R., Nawab A., Sharma H., Bhaskaran N., Ponsky L.E., Fu P., MacLennan G.T. (2020). Oxidative Stress and Antioxidant Status in High-Risk Prostate Cancer Subjects. Diagnostics.

[128] Bray F., Ferlay J., Soerjomataram I., Siegel R.L., Torre L.A., Jemal A. (2018). Global cancer statistics 2018: GLOBOCAN estimates of incidence and mortality worldwide for 36 cancers in 185 countries. CA Cancer J. Clin..

[129] Koklesova L., Liskova A., Samec M., Buhrmann C., Samuel S.M., Varghese E., Ashrafizadeh M., Najafi M., Shakibaei M., Büsselberg D. (2020). Carotenoids in Cancer Apoptosis—The Road *from Bench to Bedside* and Back. Cancers.

[130] Liskova A., Koklesova L., Samec M., Smejkal K., Samuel S.M., Varghese E., Abotaleb M., Biringer K., Kudela E., Danko J. (2020). Flavonoids in Cancer Metastasis. Cancers.

[131] Liskova A., Kubatka P., Samec M., Zubor P., Mlyncek M., Bielik T., Samuel S.M., Zulli A., Kwon T.K., Büsselberg D. (2019). Dietary Phytochemicals Targeting Cancer Stem Cells. Molecules.

[132] Abotaleb M., Liskova A., Kubatka P., Büsselberg D. (2020). Therapeutic Potential of Plant Phenolic Acids in the Treatment of Cancer. Biomolecules.

[133] Kapinova A., Kubatka P., Liskova A., Baranenko D., Kruzliak P., Matta M., Büsselberg D., Malicherova B., Zulli A., Kwon T.K. (2019). Controlling metastatic cancer: The role of phytochemicals in cell signaling. J. Cancer Res. Clin. Oncol..

[134] Liskova A., Koklesova L., Samec M., Varghese E., Abotaleb M., Samuel S.M., Smejkal K., Biringer K., Petras M., Blahutova D. (2020). Implications of flavonoids as potential modulators of cancer neovascularity. J. Cancer Res. Clin. Oncol..

[135] Samec M., Liskova A., Kubatka P., Uramova S., Zubor P., Samuel S.M., Zulli A., Pec M., Bielik T., Biringer K. (2019). The role of dietary phytochemicals in the carcinogenesis via the modulation of miRNA expression. J. Cancer Res. Clin. Oncol..

[136] Buhrmann C., Shayan P., Brockmueller A., Shakibaei M. (2020). Resveratrol Suppresses Cross-Talk between Colorectal Cancer Cells and Stromal Cells in Multicellular Tumor Microenvironment: A Bridge between In Vitro and In Vivo Tumor Microenvironment Study. Molecules.

[137] Farkhondeh T., Mehrpour O., Buhrmann C., Pourbagher-Shahri A.M., Shakibaei M., Samarghandian S. (2020). Organophosphorus Compounds and MAPK Signaling Pathways. Int. J. Mol. Sci..

[138] Farhood B., Ashrafizadeh M., Khodamoradi E., Hoseini-Ghahfarokhi M., Afrashi S., Musa A.E., Najafi M. (2020). Targeting of cellular redox metabolism for mitigation of radiation injury. Life Sci..

[139] Ashrafizadeh M., Ahmadi Z., Farkhondeh T., Samarghandian S. (2020). Autophagy regulation using luteolin: New insight into its anti-tumor activity. Cancer Cell Int..

[140] Ashrafizadeh M., Ahmadi Z., Mohammadinejad R., Afshar E.G. (2020). Tangeretin: A mechanistic review of its pharmacological and therapeutic effects. J. Basic Clin. Physiol. Pharmacol..

[141] Ashrafizadeh M., Rafiei H., Mohammadinejad R., Afshar E.G., Farkhondeh T., Samarghandian S. (2020). Potential therapeutic effects of curcumin mediated by JAK/STAT signaling pathway: A review. Phytother. Res..

[142] Ashrafizadeh M., Tavakol S., Ahmadi Z., Roomiani S., Mohammadinejad R., Samarghandian S. (2020). Therapeutic effects of kaempferol affecting autophagy and endoplasmic reticulum stress. Phytother. Res..

[143] Kubatka P., Kapinová A., Kruzliak P., Kello M., Výbohová D., Kajo K., Novak M., Chripkova M., Adamkov M., Pec M. (2015). Antineoplastic effects of Chlorella pyrenoidosa in the breast cancer model. Nutrition.

[144] Kapinova A., Stefanicka P., Kubatka P., Zubor P., Uramova S., Kello M., Mojzis J., Blahutova D., Qaradakhi T., Zulli A. (2017). Are plant-based functional foods better choice against cancer than single phytochemicals? A critical review of current breast cancer research. Biomed. Pharmacother..

[145] Kubatka P., Uramova S., Kello M., Kajo K., Kruzliak P., Mojzis J., Vybohova D., Adamkov M., Jasek K., Lasabova Z. (2017). Antineoplastic effects of clove buds (Syzygium aromaticumL.) in the model of breast carcinoma. J. Cell. Mol. Med..

[146] Kubatka P., Kello M., Kajo K., Kruzliak P., Výbohová D., Mojžiš J., Adamkov M., Fialová S., Veizerová L., Zulli A. (2017). Oregano demonstrates distinct tumour-suppressive effects in the breast carcinoma model. Eur. J. Nutr..

[147] Kubatka P., Kello M., Kajo K., Samec M., Jasek K., Vybohova D., Uramova S., Líšková A., Sadlonova V., Koklesova L. (2020). Chemopreventive and Therapeutic Efficacy of Cinnamomum zeylanicum L. Bark in Experimental Breast Carcinoma: Mechanistic In Vivo and In Vitro Analyses. Molecules.

[148] Kubatka P., Kello M., Kajo K., Samec M., Liskova A., Jasek K., Koklesova L., Kuruc T., Adamkov M., Smejkal K. (2020). *Rhus coriaria* L. (Sumac) Demonstrates Oncostatic Activity in the Therapeutic and Preventive Model of Breast Carcinoma. Int. J. Mol. Sci..

[149] Jasek K., Kubatka P., Samec M., Liskova A., Smejkal K., Vybohova D., Bugos O., Biskupska-Bodova K., Bielik T., Zubor P. (2019). DNA Methylation Status in Cancer Disease: Modulations by Plant-Derived Natural Compounds and Dietary Interventions. Biomolecules.

[150] Samec M., Liskova A., Koklesova L., Mestanova V., Franekova M., Kassayova M., Bojkova B., Uramova S., Zubor P., Janikova K. (2019). Fluctuations of Histone Chemical Modifications in Breast, Prostate, and Colorectal Cancer: An Implication of Phytochemicals as Defenders of Chromatin Equilibrium. Biomolecules.

[151] Hano C., Tungmunnithum D. (2020). Plant Polyphenols, More than Just Simple Natural Antioxidants: Oxidative Stress, Aging and Age-Related Diseases. Medicines.

[152] Avula S., Parikh S., Demarest S., Kurz J., Gropman A. (2014). Treatment of Mitochondrial Disorders. Curr. Treat. Options Neurol..

[153] Parikh S., Saneto R., Falk M.J., Anselm I., Cohen B.H., Haas R., The Mitochondrial Medicine Society (2009). A modern approach to the treatment of mitochondrial disease. Curr. Treat. Options Neurol..

[154] Gibellini L., Bianchini E., De Biasi S., Nasi M., Cossarizza A., Pinti M. (2015). Natural Compounds Modulating Mitochondrial Functions. Evid.-Based Complement. Altern. Med..

[155] Kicinska A., Jarmuszkiewicz W. (2020). Flavonoids and Mitochondria: Activation of Cytoprotective Pathways?. Molecules.

[156] Lee H.A., Kim J.E., Sung J.E., Bin Yun W., Kim D.S., Lee H.S., Hong J.T., Hwang D.Y. (2018). Asparagus cochinchinensis stimulates release of nerve growth factor and abrogates oxidative stress in the Tg2576 model for Alzheimer’s disease. BMC Complement. Altern. Med..

[157] Godoy J.A., Lindsay C.B., Quintanilla R.A., Carvajal F.J., Cerpa W., Inestrosa N.C. (2017). Quercetin Exerts Differential Neuroprotective Effects Against H2O2 and Aβ Aggregates in Hippocampal Neurons: The Role of Mitochondria. Mol. Neurobiol..

[158] Malvajerd S.S., Izadi Z., Azadi A., Kurd M., Derakhshankhah H., Zadeh M.S., Javar H.A., Hamidi M. (2019). Neuroprotective Potential of Curcumin-Loaded Nanostructured Lipid Carrier in an Animal Model of Alzheimer’s Disease: Behavioral and Biochemical Evidence. J. Alzheimer’s Dis..

[159] Garcia-Moreno J.C., De La Riva M.P., Martínez-Lara E., Siles E., Cañuelo A. (2019). Tyrosol, a simple phenol from EVOO, targets multiple pathogenic mechanisms of neurodegeneration in a C. elegans model of Parkinson’s disease. Neurobiol. Aging.

[160] Baroli B., Loi E., Solari P., Kasture A., Moi L., Muroni P., Kasture S., Setzu M.D., Liscia A., Zavattari P. (2019). Evaluation of oxidative stress mechanisms and the effects of phytotherapic extracts on Parkinson’s disease Drosophila PINK1 B9 model. FASEB J..

[161] Koklesova L., Liskova A., Samec M., Qaradakhi T., Zulli A., Smejkal K., Kajo K., Jakubikova J., Behzadi P., Pec M. (2020). Genoprotective activities of plant natural substances in cancer and chemopreventive strategies in the context of 3P medicine. EPMA J..

[162] Liskova A., Stefanicka P., Samec M., Smejkal K., Zubor P., Bielik T., Biskupska-Bodova K., Kwon T.K., Danko J., Büsselberg D. (2020). Dietary phytochemicals as the potential protectors against carcinogenesis and their role in cancer chemoprevention. Clin. Exp. Med..

[163] Finsterer J. (2004). Mitochondriopathies. Eur. J. Neurol..

[164] Yeghiazaryan K., Flammer J., Golubnitschaja O. (2010). Predictive molecular profiling in blood of healthy vasospastic individuals: Clue to targeted prevention as personalised medicine to effective costs. EPMA J..

[165] Bubnov R., Polivka J., Zubor P., Konieczka K., Golubnitschaja O. (2017). “Pre-metastatic niches” in breast cancer: Are they created by or prior to the tumour onset? “Flammer Syndrome” relevance to address the question. EPMA J..

[166] Avishai E., Yeghiazaryan K., Golubnitschaja O. (2017). Impaired wound healing: Facts and hypotheses for multi-professional considerations in predictive, preventive and personalised medicine. EPMA J..

[167] Qian S., Golubnitschaja O., Zhan X. (2019). Chronic inflammation: Key player and biomarker-set to predict and prevent cancer development and progression based on individualized patient profiles. EPMA J..

[168] Baban B., Golubnitschaja O. (2017). The potential relationship between Flammer and Sjögren syndromes: The chime of dysfunction. EPMA J..

[169] Scozzi D., Cano M., Ma L., Zhou D., Zhu J.H., O’Halloran J.A., Goss C.W., Rauseo A.M., Liu Z., Sahu S.K. (2021). Circulating mitochondrial DNA is an early indicator of severe illness and mortality from COVID-19. JCI Insight.

[170] Chaari L., Golubnitschaja O. (2020). Covid-19 pandemic by the “real-time” monitoring: The Tunisian case and lessons for global epidemics in the context of 3PM strategies. EPMA J..

[171] Yeghiazaryan K., Cebioglu M., Braun M., Kuhn W., Schild H.H., Golubnitschaja O. (2011). Noninvasive subcellular imaging in breast cancer risk assessment: Construction of diagnostic windows. Pers. Med..

[172] Lu M., Zhan X. (2018). The crucial role of multiomic approach in cancer research and clinically relevant outcomes. EPMA J..

[173] Goldstein E., Yeghiazaryan K., Ahmad A., Giordano F.A., Fröhlich H., Golubnitschaja O. (2020). Optimal multiparametric set-up modelled for best survival outcomes in palliative treatment of liver malignancies: Unsupervised machine learning and 3 PM recommendations. EPMA J..

[174] Bubnov R., Babenko L., Lazarenko L., Kryvtsova M., Shcherbakov O., Zholobak N., Golubnitschaja O., Spivak M. (2019). Can tailored nanoceria act as a prebiotic? Report on improved lipid profile and gut microbiota in obese mice. EPMA J..

